# Broad Spectrum Anticancer Activity of Myo-Inositol and Inositol Hexakisphosphate

**DOI:** 10.1155/2016/5616807

**Published:** 2016-10-04

**Authors:** Mariano Bizzarri, Simona Dinicola, Arturo Bevilacqua, Alessandra Cucina

**Affiliations:** ^1^Department of Experimental Medicine, Sapienza University of Rome, Viale Regina Elena 324, 00161 Rome, Italy; ^2^Systems Biology Group Lab, Sapienza University of Rome, Rome, Italy; ^3^Department of Clinical and Molecular Medicine, Sapienza University of Rome, Viale Regina Elena 336, 00161 Rome, Italy; ^4^Department of Surgery “Pietro Valdoni”, Sapienza University of Rome, Via A. Scarpa 14, 00161 Rome, Italy; ^5^Department of Psychology, Section of Neuroscience, Sapienza University of Rome, Via dei Marsi 78, 00185 Rome, Italy; ^6^Azienda Policlinico Umberto I, Viale del Policlinico 155, 00161 Rome, Italy

## Abstract

Inositols (myo-inositol and inositol hexakisphosphate) exert a wide range of critical activities in both physiological and pathological settings. Deregulated inositol metabolism has been recorded in a number of diseases, including cancer, where inositol modulates different critical pathways. Inositols inhibit pRB phosphorylation, fostering the pRB/E2F complexes formation and blocking progression along the cell cycle. Inositols reduce PI3K levels, thus counteracting the activation of the PKC/RAS/ERK pathway downstream of PI3K activation. Upstream of that pathway, inositols disrupt the ligand interaction between FGF and its receptor as well as with the EGF-transduction processes involving IGF-II receptor and AP-1 complexes. Additionally, Akt activation is severely impaired upon inositol addition. Downregulation of both Akt and ERK leads consequently to NF-kB inhibition and reduced expression of inflammatory markers (COX-2 and PGE2). Remarkably, inositol-induced downregulation of presenilin-1 interferes with the epithelial-mesenchymal transition and reduces Wnt-activation, *β*-catenin translocation, Notch-1, N-cadherin, and SNAI1 release. Inositols interfere also with the cytoskeleton by upregulating Focal Adhesion Kinase and E-cadherin and decreasing Fascin and Cofilin, two main components of pseudopodia, leading hence to invasiveness impairment. This effect is reinforced by the inositol-induced inhibition on metalloproteinases and ROCK1/2 release. Overall, these effects enable inositols to remodel the cytoskeleton architecture.

## 1. Introduction

Inositol (myo-Ins) and its phosphate metabolites exert a wide range of critical activities in both physiological and pathological settings. Indeed, deregulation of inositol metabolism has been extensively investigated in several illnesses, including neurological disorders [1], polycystic ovary syndrome [2], and metabolic diseases [3].

In the wake of the renewed interest for inositol phosphates (InsPs) and other inositol-based compounds, studies on the anticancer properties of both inositol hexakisphosphate (InsP6) and myo-Ins have gained momentum during the last decades. InsP6 inhibits growth and invasiveness of a number of cancer types, while both InsP6 and myo-Ins have been demonstrated to display significant chemopreventive effects both* in vitro* and* in vivo*. Furthermore, inositols have been involved in modulating unexpected processes, including mRNA transcription, chromatin remodeling, cytoskeleton configuration, and p53 activity, just to mention a few. Therefore, these new findings prompted reassessing under a new light the putative role of both myo-Ins and InsP6 in carcinogenesis.

## 2. Epidemiology: Diet and Cancer

Since the early eighties [4], it has been recognized that wide variation in cancer incidence among different countries around the world can primarily be ascribed to environmental factors, among which diet is likely the most important [5]. This evidence is very strong for some cancers, like breast, prostate, and colon tumors, where differences in tumor incidence across countries have been mainly ascribed to their respective dietary habits [6]. Those data provided the rationale for the so-called “fiber hypotheses” [7], for which grains refining and lack of dietary fiber may have a “causative” role in colon and breast carcinogenesis [8]. Several investigations led support to this hypothesis [9], even if some inconsistencies have been recorded [10]. Yet, such association is probably a too simplistic one, given that fibers could not be the sole putative preventive factor. Indeed, colon cancer incidence has been shown to differ significantly among groups consuming approximately the same amount of fibers [11]. These findings indicate that to assess the correlation between diet and cancer properly we should evaluate the consumption of specific components rather than focusing on the overall fiber intake. Both epidemiological and molecular investigations have indeed provided valuable data suggesting that distinct dietary components may exert specific anticancer activities. Among those nutrients, compelling evidence gathered to date has evidenced that lignans, polyphenolic acids, stilbenes, bioflavonoids, phytic acid, and inositols exert unquestionable anticancer effects [12, 13]. Moreover, it has been observed that only the consumption of fibers with high content of phytic acid is inversely correlated with colon cancer [14].

## 3. Inositol and Inositol Hexakisphosphate

InsP6 is contained mainly in cereals, legumes, and oilseed [15]. The presence of a phosphate group in positions 1, 2, and 3 (axial-equatorial axis) confers unique properties to it as this configuration provides a specific chelating capacity regarding polyvalent cations, including iron and other potentially toxic elements (Ni, Zn, Cu, and even Uranium) [16, 17]. This property makes InsP6 an excellent chelator of many potentially harmful trace elements that have been shown to cause deleterious effects in humans [18]. Moreover, InsP6 capacity in blocking hydroxyl radical formation makes phytic acid a strong physiological antioxidant [19]. Insofar as InsP6 is often referred to as an antinutrient [20] responsible for iron deficiencies mostly in underdeveloped countries, it should be emphasized that InsP6 displays its antinutritional effects only when the diet is already deprived of trace elements [21].

Dietary InsP6 is mainly digested in the gut by bacterial phytases and phosphatase [22], thus releasing myo-Ins and other inositol phosphates (InsPs). Yet, a variable fraction of dietary IP6 is directly absorbed as such and can be recovered in plasma and urine [23], even if this assumption has been subject of controversy [24, 25].

A mixed western diet provides the human adult with approximately 1 g of total inositol per day [26]. No requirement for dietary inositol in man has been determined, even if physiological needs could be highly variable depending on the person's age, the long-term use of antibiotics, or the regular consumption of coffee [27]. How appropriate the bioavailability of myo-Ins and InsP6 in western alimentary regimens is still constitutes a matter of debate [28, 29]. By considering that from the ‘70s many foods have been processed to remove phytic acid (owing to its alleged antinutritional effects) it may be surmised that in western countries low-vegetable consumers may suffer from a relative deficiency of myo-Ins due to the reduced content of both myo-Ins and phytic acid in the diet. Furthermore, assessment of myo-Ins requirements is further complicated by the fact that a significant amount of inositol is endogenously synthesized from glucose. Glucose-6-phosphate is isomerized by D-3-myo-inositol-phosphate synthase (MIPS1 encoded by the ISYNA1 gene) to yield inositol-3-phosphate (Ins3P) and then converted by inositol monophosphatase-1 (IMPA-1) into free myo-Ins [30]. Both enzymes are inducible in response to the specific tissue requirements, thus explaining why myo-Ins concentrations differ so greatly among different tissues and physiological conditions [31].

After cellular internalization through endocytosis InsP6 is partially dephosphorylated yielding myo-inositol and inositol phosphates, mainly InsP5 [32]. However, free myo-Ins is actively transported into cells by a means of complex transport system. Within cells myo-Ins is converted into inositol phospholipids (phosphatidylinositol, PI, and phosphatidylinositol phosphate(s), PIP_(s)_), inositol-glycans (IPGs), inositol phosphates (InsPs, including InsP6), and pyrophosphates (PP-IPs), according to a complex network extensively reviewed elsewhere [33, 34].

## 4. Molecular Mechanisms of Action

### 4.1. Cell Cycle Control and Apoptosis

Several studies have investigated the inhibitory activity of InsP6 on cancer cells from both animals and humans. Results are unambiguous and show that InsP6 induces G_1_ phase arrest and abridges S phase of cancer cells, mainly by modulation of cyclins, upregulation of p53, p57, p27^Kip1^, and p21^WAF/CIP1^, and downregulation of phosphorylated pRb [35–37]. The family of pRB subunits (pRB/p107 and pRB2/p130) inhibits the cell cycle progression by forming complexes with E2F in the G0 phase. InsP6, by increasing the hypophosphorylated form of pRB, increases the pRB/E2F complexes formation, thus blocking further progression along the cell cycle [38]. Consequently, in InsP6-treated cancer cells, a significant downregulation of genes involved in cell cycle advancement (like c-*myc*, cyclin H, and FUSE) and an upregulation of those activated during cycle inhibition (CSK2, p57, and Id-2) have been observed [38]. Inhibition of breast cancer cell proliferation occurs independently from the estrogen receptor (ER) status, as it was achieved in both ER negative (MDA-MB-231) and positive (MCF-7) cells [39]. Similar results have been obtained in other cancers, even though subtle differences have been recorded among different cell lines [40, 41]. For instance, when leukemia cells were treated with InsP6, only some cell lines (A230 and K562) were arrested in G_2_/M phase, while other cell lines (including CD34^+^ from myelogenous leukemia patients) were committed to apoptosis [42]. Early studies have suggested that InsP6 effect is rather cytostatic than cytotoxic [43, 44]. However, further investigation demonstrated that InsP6 had unequivocal apoptotic effects on both solid and haematogenous tumors. Indeed, InsP6 has been shown to trigger programmed cell death both* in vitro* and* in vivo* [45] in numerous cancer cell lines including Kaposi's sarcoma [46] and prostate, breast, cervical, pancreas, melanoma [47], and colon cancer [48–50]. This apoptotic effect is frequently associated with growth inhibition [35, 51] and ascertaining whether both effects occur independently from each other still needs to be investigated. Additionally, InsP6 has been shown to synergize with both doxorubicin and tamoxifen in inhibiting breast cancer growth, namely, in drug-resistant cancer cell lines [52]. This result implies that InsP6 may counteract drug resistance frequently displayed by tumor cells and should therefore be considered a useful adjunct in delivering conventional anticancer drugs. On the contrary, myo-Ins has been shown to have only a minimal proapoptotic activity and to induce a mild decrease in growth proliferation in colon, breast, soft tissue, and lung tumors [53]. Yet, myo-Ins is able to significantly synergize with InsP6, both* in vitro* and* in vivo*, in inducing cancer inhibition [54].

However, some results hint at a more subtle and complex role for inositol and its phosphate derivatives. In some circumstances, instead of apoptosis or growth inhibition, cell differentiation occurs after InsP6 treatment. Induction of differentiation in human erythroleukemia cells was preliminarily evidenced following InsP6 and subsequently in several other cancers, including rhabdomyosarcoma and breast, colon, and prostate tumors [55–57]. Why cancer cells respond so differently following InsP6 administration is poorly understood. It can be hypothesized that other factors, namely, other inositol phosphate derivatives, may participate in such processes, thereby driving the final output into diverse fates [58]. Yet, the contribution of context-dependent cues in modulating InsP6 effects cannot be discarded.

### 4.2. The p53 Network

Inhibition of cell proliferation and induction of apoptosis have been recorded in numerous cancer cell lines after InsP6 treatment. A crucial factor in both issues is represented by p53 activity and the subsequent selective pathways triggered downstream of p53. InsP6 increases p53 levels severalfold at both mRNA and protein levels [47, 59]. However, consistent data suggest that p53 is not mandatory for triggering InsP6-related effects, as apoptosis and inhibition of cell growth have been both observed in cancer cells lacking p53 [60]. On the contrary, p27 and p21 should be considered as essential molecular target of InsP6, given that the simultaneous knockdown of both p21 and p27 completely abrogates the anticancer effects of InsP6 [51]. By analogy, myo-Ins has been proven to reduce lung cancer incidence in mouse lacking p53 and treated with N-nitrosomethylurea [61]. Yet, a very recent paper demonstrated that oral myo-Ins does not suppress cancer development in p53 knockout mice [62], while evidence about the proapoptotic effect of myo-inositol is still inconclusive even in presence of p53. Thereby the question is still open and further studies are warranted to understand whether p53 activity is effectively required in mediating anticancer effects displayed by both InsP6 and myo-Ins. Downstream of p53 InsP6 has been demonstrated to reduce prosurvival factors and to upregulate caspases and other components of the proapoptotic BCL-2 family [63–66]. Furthermore, InsP6 has been shown to inhibit NF-kB activity in different cancers [67, 68]. NF-kB is a pivotal factor involved in fostering both survival pathways and the epithelial-mesenchymal transition (EMT). Therefore, targeting NF-kB is currently deemed a promising approach in cancer management. In prostate carcinoma, constitutive activation of NF-kB is inhibited by InsP6 [69], while in HeLa cells phytic acid prevents nuclear translocation of NF-kB and NF-kB-luciferase transcription activity [49]. In Caco-2 colon cancer cells, InsP6-mediated NF-kB inhibition is likely to occur through the block of the p65 subunit of NF-kB and its inhibitor IkBa [50].

As observed with other natural compounds (grape seed extracts, melatonin), the apoptotic effect triggered by inositol derivatives seems to be specific for cancer cells, given that both InsP6 and myo-Ins did not promote apoptosis in normal cells. Moreover, a “paradoxical” antiapoptotic effect of InsP6 has been noticed in normal cells exposed to iron-induced apoptosis [70]. Therefore, why normal and cancerous cells respond differently to both InsP6 and myo-inositol still deserves to be explained in detail.

### 4.3. Inhibition of the PI3K/Akt Pathway

The PI3K/Akt pathway is undoubtedly a pivotal hub upstream the activation of survival pathways, including the activation of Wnt and NF-kB [71]. PI3K triggers activation of Akt kinases through direct binding to the pleckstrin homology domain and the subsequent phosphorylation of Akt at two conserved residues. Hence, activated Akt modulates the function of numerous substrates involved in the regulation of cell survival, cell cycle progression, and cellular growth, eventually enabling cancer cells to become more aggressive [72]. These findings make the PI3K/Akt pathway one of the most attractive targets for therapeutic intervention. It is therefore worth noting that both InsP6 and myo-Ins significantly reduce PI3K expression (at both mRNA and protein levels) [73] and Akt activation by inhibiting its phosphorylation [74, 75]. InsP6 impairs directly PI3K activity and thus the PI3K-dependent activation of the tumor promoter-induced AP-1, as well as the phosphorylation-dependent activation of ERK [75]. Inhibition of PI3K activity and subsequent blocking of PKC and mitogen-activated kinases (MAPK) have been so far documented by several* in vitro* [76–78] and* in vivo* chemopreventive studies [79, 80]. Additionally, InsP6 interacts with clathrin-associated protein complex-2 and inhibits PI3K, ERK, and MAPK activation, thus impairing ErbB1 endocytosis and ligand-induced Shc phosphorylation [81]. Given that PI3K/Akt pathway activity is mandatorily required for triggering EMT, blocking PI3K would hinder the transformation of cancer cells into a more aggressive phenotype. Indeed, breast cancer cells treated* in vitro* with myo-Ins showed increased E-cadherin, downregulation of metalloproteinase-9, and redistribution of *β*-catenin behind cell membrane, while motility and invading capacity were severely inhibited [75]. Those changes were associated with a significant downregulation of PI3K/Akt activity, leading to a decrease in downstream signaling effectors: NF-kB, COX-2, and SNAI1. Moreover, myo-Ins decreases presenilin-1 (PS1) levels and inhibits its activity, thus leading to lowered Notch-1 release and SNAI1 levels. Furthermore, inositol-treated cells underwent profound cytoskeleton remodeling [75]. Overall, these data indicated that myo-Ins inhibits the principal molecular pathway supporting EMT in cancer cells.

### 4.4. Inhibition of Invasiveness and Motility

The ability of cancer to metastasize relies primarily on the invasiveness and increased motility of tumor cells. It is therefore worth noting that, by blocking EMT, myo-Ins significantly hampers both motility and invasiveness of breast cancer cells. This effect is likely to be ascribed to cytoskeleton remodeling and to the concomitant inhibition of metalloproteinases (MMPs) release [75]. Similarly, InsP6 significantly reduces the number of lung metastatic colonies in a mouse metastatic tumor model [82], while in MDA-MB-231 breast cancer cells this effect is mediated by reduced adhesion and MMPs release [83, 84].

### 4.5. Wnt Signaling and Anti-Inflammatory Effects

Activation of the Wnt/*β*-catenin pathway occurs in several cancers. Overexpression of the Wnt ligand, usually in association with deregulated *γ*-secretase activity, may lead to deregulated expression and redistribution of *β*-catenin and of several molecular factors belonging to the so-called inflammatory pathway, like COX-2 and PGE2 [85]. Increased expression of the aforementioned molecules has been demonstrated to be associated with carcinogenesis in numerous tissues, chiefly in colon cancer [86]. It is of high relevance that InsP6 downregulates both* in vitro* and* in vivo* the Wnt pathway* via* *β*-catenin inhibition, thus significantly reducing COX-2 at both the mRNA and protein levels [87]. Eventually, this study demonstrated that InsP6 administration markedly suppressed in a dose-dependent manner the incidence of cancer in male Sprague Dawley rats when compared to controls. Moreover, InsP6 counteracts the proliferative response following inflammatory injury by inhibiting cyclin D1 and histone H3 expression [88].

In breast cancer cells, myo-Ins has been proven to downregulate both NF-kB and COX-2, while relocating *β*-catenin behind cell membrane [76]. Such inhibitory effects on inflammatory markers may not be confined to epithelial cells but should also probably involve the surrounding microenvironment. Indeed, both InsP6 and myo-Ins have been demonstrated to prevent pulmonary fibrosis, breast density, and chronic inflammatory damage, likely by influencing the crosstalk among cells and their milieu [89–91]. Given that TGF*β*-1 released by both fibroblasts and epithelial cells is a profibrogenic factor regulating the balance between matrix-degrading metalloproteinases and their inhibitors [92], it is quite exciting that myo-Ins has been demonstrated to modulate the expression of both TGF*β* and its receptors. Indeed, myo-Ins mitigates colonic epithelium inflammation as well as inflammatory consequences on colon stromal cells during microbial infections [93, 94]. Furthermore, InsP6 has been shown to exert valuable effects on fibroblasts by blocking the syndecan-4 dependent focal adhesion and microfilament bound [95]. Syndecan-4 is a heparan sulphate proteoglycan embedded into cellular membranes, where it regulates cell-matrix interactions by interfering with cytoskeleton proteins and integrins. Indeed, in human mammary cancer cell lines, cell adhesion to extracellular matrix was decreased after InsP6 treatment [84]. Moreover, syndecan binds to the fibroblast growth factor (FGF), fostering its coupling with the FGF receptor. InsP6 disrupts such interaction, thus inhibiting the FGF-based signaling [96]. Inositol-related effects on the cell* milieu* also involve modulation of angiogenesis. Formation of new blood vessels is required for sustaining cancer growth and invasiveness. Disruption of the structural relationships among cancer cells and their microenvironment promotes neoangiogenesis, mainly through the release of vascular endothelial growth factor (VEGF). InsP6 negatively modulates VEGF release from tumor cells [45] and impairs endothelial cells growth [97]. Likely, VEGF reduced synthesis may be due to InsP6-mediated inhibition on PI3K/Akt and MAPK/ERK pathways [82], given that both of them are deemed to modulate VEGF upregulation [98, 99]. Additionally, the synergistic activity of hypoxia and IGF-II increases VEGF mRNA expression and upregulates HIG-1 protein that, in turn, reinforces VEGF release [100]. Given that InsP6 has been shown to antagonize IGF-II activity by inhibiting the IGF-II receptor binding [101], it is likely that some InsP6 antiangiogenic effects can be ascribed to this mechanism.

Overall, these data suggest that inositol and its phosphate derivatives exert complex biological functions involving both cells and stromal factors. Yet, given the entrenched correlations occurring among cells and microenvironment during carcinogenesis [102, 103] the stromal effects of both InsP6 and myo-Ins deserve to be still fully investigated.

### 4.6. Anticancer Activity through Insulin Modulation

Myo-inositol and its isomer D-chiro-inositol (D-chiro-Ins) participate in both insulin and glucose metabolisms, and deregulated myo-Ins metabolism has been documented in several conditions associated with diabetes or insulin resistance [3]. Indeed, low levels of inositol have been observed in biological fluids and insulin target tissues (muscle, liver, and fat), frequently associated with excessive myo-Ins renal excretion, while low intracellular levels of myo-Ins have been detected in insulin insensitive tissues [104]. When insulin binds to its receptor, two distinct inositol-phosphoglycans (IPGs), incorporating either myo-Ins or D-Chiro-Ins (IPG-A and IPG-P), are released by insulin-stimulated hydrolysis of glycosyl-phosphatidylinositol lipids located on the outer leaflet of the cell membrane. IPGs affect intracellular metabolic processes, namely, by activating key enzymes controlling the oxidative and nonoxidative metabolism of glucose and acting as insulin-mimetic when administered* in vivo* in normal or diabetic rats [105]. Glycan derivatives of inositol significantly reduce insulin resistance and promote appropriate glucose metabolism [106]. Given that myo-Ins may efficiently counteract insulin resistance and its metabolic complications [107], it is tempting to speculate that it may also prevent IGF-1 increase associated with insulin resistance. As both insulin resistance and IGF-1 are linked to increased cancer risk [108], it is conceivable that myo-Ins modulation of insulin activity may efficiently contribute to reducing cancer risk. Indeed, InsP6 has been already shown to inhibit the IGF-1 receptor pathway-mediated sustained growth in cancer cells [85]. Moreover, cancer cells are featured by a glycolytic metabolomic fingerprint, thought to confer a “proliferative advantage” during the neoplastic development [109]. It is therefore tempting to speculate if inositol addition can antagonize cancer development by normalizing glucose metabolism in cancer cells, another matter that eventually still needs to be fully investigated.

### 4.7. Antioxidant and Other Effects

Myo-Ins displays a moderate antioxidant activity, while InsP6 is among the strongest antioxidants present in nature. By chelating polyvalent cations, InsP6 and myo-Ins suppress Fenton's reaction and the consequent release of hydroxyl radicals [110]. In biological tissues InsP6 has been shown to inhibit xanthine oxidase [111] and reactive oxygen species production, thus dramatically inhibiting the free radical-based damage occurring in cells and tissues following inflammation, hypoxia, or exposition to radiation injury [91, 112, 113]. Myo-Ins counteracts oxidative damage in fish exposed to environmental stresses [114] and significantly inhibits systemic markers of oxidative stress in gynecological patients [115]. InsP6 scavenges superoxide radicals* in vitro* and* in vivo*, thus preventing formation of ADP-iron-oxygen complexes that trigger lipid peroxidation [116]. Indeed, inhibition of lipid peroxidation has been documented in animals after InsP6 administration [117, 118]. As increases in both ROS and lipid peroxidation have been associated with cancer development, it has been hypothesized that some anticancer chemopreventive effects displayed by InsP6 and myo-Ins could therefore be ascribed to their antioxidant capability. However, as recorded for other natural compounds, the antioxidant property of inositol is strictly context-dependent as, under specific conditions, both myo-Ins and InsP6 may increase free radical production [119].

## 5. Effects on the Immune Function

Even if it is still limited, current evidence suggests that inositols may play an appreciable regulatory activity on immune function* in vitro* and* in vivo*. Inositol hexakisphosphate and myo-Ins enhance NK activity in mice treated with 1,2-dimethylhydrazine (DMH), a colon carcinogen, which also significantly reduces NK function [120, 121]. In this model, InsP6 also reverses tumor induction, decreases cancer-related death, and specifically boosts NK cytotoxicity in a dose-dependent manner. As previously observed in other studies, the association of InsP6 and myo-Ins displays synergistic effects, given that significantly better results were observed in animals treated with a combination of both [120]. InsP6 acts as a neutrophil priming agent and it upregulates several neutrophil functions, including enhancing superoxide production and phagocytosis [119]. Additionally, InsP6 modulates a number of inflammatory markers, namely, involving IL-8 release by stimulated neutrophils [119]. InsP6 also modulates the transcription genes for TNF by decreasing it and its receptors in colon cancer cells [122]. This downregulating effect of InsP6 on inflammatory processes is mirrored by the aforementioned inhibitory activity displayed by myo-Ins on several inflammatory pathways (COX-2 and PGE2) [76]. Therefore, it seems that both inositols exert inhibitory control on the activation of the inflammatory pathways, which are frequently upregulated during carcinogenesis.

## 6. Chemopreventive and Therapeutic Efficacy in Animal Studies

The chemopreventive as well as the therapeutic activity* in vivo* of InsP6 has been documented by an impressive body of studies. Exogenous administration of InsP6 in drinking water, one or two weeks after azoxymethane induced carcinogenesis, prevents the onset of colon cancer in Fisher rats [123]. Preventive activity was also observed when InsP6 was added in higher concentration 5 months later after the carcinogenic stimulation [124]. Inositol hexakisphosphate can indeed prevent even the formation of aberrant colon crypts, thought to be the histological precursor of the neoplastic transformation [125].

On the other hand, inositol hexakisphosphate may potentiate the anticancer effects of conventional chemotherapy in preventing the successful development of cancer implants. Indeed, the administration of liposomes containing both InsP6 and irinotecan (CPT-11) showed higher efficacy in inhibiting the viability and the growth of colon tumor xenografts in mouse when compared to single compounds alone [126]. InsP6 chemopreventive activity is not restricted to the gastrointestinal tract, as it has been shown that inositols may efficiently counteract the carcinogenic effect of chemicals on breast tissue. Breast tumor incidence after exposure to 7,12-dimethylbenz[*a*]anthracene or N-methylnitrosourea is significantly reduced in animals treated with InsP6. Similarly, InsP6 dramatically reduces by almost 64% the burden of implanted DU-145 prostate cancer [35] as well as the growth of transgenic adenocarcinoma of the prostate in mouse [127, 128]. Similarly, inositol hexakisphosphate inhibits growth and induces G_1_ arrest and apoptotic death of androgen-dependent human prostate carcinoma LNCaP cells [40]. Namely, in a transgenic mouse model of prostate carcinoma (TRAMP), orally administered InsP6 has been able to inhibit cancer progression at prostatic intraepithelial neoplasia stage and strongly reduced the incidence of adenocarcinoma (prostatic intraepithelial neoplasia/adenocarcinoma, 75 : 25% in the InsP6 group versus 39 : 61% in the control group) [127]. These findings evidence the chemopreventive efficacy of oral administered InsP6* in vivo* as well as its safety.

Additionally, InsP6 or myo-Ins has been shown to induce the regression of other different types of cancer, like rhabdomyosarcoma, liver cancer [129], soft tissue [130], and fibrosarcoma [43, 83, 131]. Skin tumorigenesis induced by chemical compounds [44] or by physical factors (i.e., UVB) [68] was also demonstrated to be significantly diminished by InsP6 administration. Both InsP6 and myo-Ins reduced the incidence and growth of lung tumors chemically induced in mice [132]. Indeed, dietary inositol has been shown to inhibit lung tumorigenesis in female A/J mice exposed to the carcinogen benzo(*α*)-pyrene or 4-(methylnitrosamino)-1-(3-pyridyl)-1-butanone: myo-Ins was also effective in the postinitiation phase and when given for short periods of time before, during, and immediately after carcinogen exposure [133, 134]. Overall, myo-Ins anticancer efficacy was proven to be lower when compared to InsP6. However, it is noticeable that myo-Ins potentiates significantly the antitumor effects displayed by InsP6* in vivo* [135, 136].

## 7. Chemopreventive and Therapeutic Efficacy in Human Clinical Trials

Both myo-Ins and InsP6 are safe, even when administered at high doses, as assessed by several clinical trials performed in cancer patients as well as in humans suffering from other diseases (mostly with gynecological diseases like PCOS). Mild side effects (mostly represented by nausea or diarrhea) are reported in a small fraction of subjects, only for doses up to 12 g/day (reviewed in [137]).

On the other hand, InsP6 has been demonstrated to exert valuable anticancer effects even* in vivo* when administered to cancer patients. An antitumor activity has been observed in advanced colon cancer patients, where InsP6 plus myo-Ins treatment is associated with appreciable reduction in tumor burden and improved quality of life. Moreover, if inositols were added along with conventional chemotherapy, colon cancer patients experienced significantly less side effects than controls, as reported in a pilot study [138]. Furthermore, prolonged survival and better quality of life have been obtained in some anecdotal cases of breast and lung cancer patients treated with InsP6 and myo-Ins [139–141]. Again, in a prospective, randomized study, InsP6 and myo-Ins ameliorate the responsiveness to chemotherapy in breast cancer patients and markedly reduce the burden of side effects [142]. As previously noticed in animal studies, myo-Ins has been demonstrated to exert a significant chemopreventive activity also in human beings [143]. A study enrolling 26 smokers showed that myo-Ins in a daily dose up to 18 g/p.o. is safe and well tolerated, while inducing a significant regression of individual pulmonary dysplastic lesions (91% in the inositol-treated group versus 48% in control group) in a sample of heavy smoker individuals [144]. A significant increase in a genomic signature of PI3K pathway activation has been documented in the cells of the bronchial airway of patients with dysplastic lesions, thus suggesting that PI3K is activated in the proximal airway before tumorigenesis. Treatment with myo-Ins is able to induce a marked regression of both dysplastic lesions and PI3K activity. Such preliminary findings have been subsequently established by two other papers [79, 80]. Unfortunately, as these trials have been carried out on very small samples of patients, no firm conclusions can be drawn from them.

Overall, those data represent, at best, only a promising preliminary hint, seldom emerging from anecdotal observations. Indeed, a number of critical factors actually limit the clinical relevance of the available results.

First, no extensive, randomized trials have been done till now. Pilot studies are potentially flawed by the reduced number of enrolled patients and (with some exceptions) the lack of randomization. Well-designed clinical studies are thereby required to evaluate, if any, the different responsiveness among men/women and the diverse sensitivity of solid/hematological cancers when treated with inositol(s). Due to the hypothetical mechanisms of inositol action, these surveys would require an extended period of observation and larger patient samples than those studied till now. That remark applies also to chemopreventive studies. Even if there are no ongoing or planned randomized clinical trials with either InsP6 or myo-Ins, a recent clinical study promoted by the NIH [145] showed no benefit associated with myo-Ins supplementation in heavy smokers carrying bronchial dysplasia. Yet, even this survey is biased by the limited number of subjects (38 in the myo-Ins arm versus 36 placebo-treated controls) entering the study.

Second, clinical studies should be aimed at recording not only the response in terms of cancer changes but also the concomitant modification in metabolic/endocrine milieu. Indeed, an almost entirely overlooked field of investigation is represented by the involvement of inositol(s) in estrogen modulation. This is a potentially outstanding issue, as inositol(s) have been shown to modulate aromatase activity as well as a number of circulating hormones (including insulin, FSH, and LH). Studies performed in women affected by PCOS have shown that aromatase activity and the release of gonadotropin-releasing factors (LH and FSH) and of numerous other hormones (including insulin, prolactin, and testosterone) are significantly modulated by inositol addition [146]. It is therefore tempting to speculate that such mechanism may also participate in triggering inositol-related anticancer effects on endocrine responsive tumors (especially breast and prostate cancer).

Third, clinical benefit ensured by the addition of InsP6 and/or myo-Ins to conventional chemotherapy may be ascribed to indirect physiological effects rather than to a “direct” anticancer effect. As previously outlined, both InsP6 and myo-Ins modulate a number of proinflammatory pathways by targeting few components of cancer stroma (fibroblasts and density of the surrounding matrix). Modulation of cancer stroma, in association with the inositol-based effect on the cytoskeleton, may efficiently contribute to reframing the functional architecture of cancer microenvironment [103], thus leading to a plethora of unexpected consequences, ultimately ending up into an inhibition of cancer growth.

Inositol(s) may indeed modulate the antioxidant/prooxidant balance, as well as the patient metabolomic fingerprint (downregulation of insulin levels, improved glucose utilization through the oxidative cycle, and inhibition of lipogenesis). Such effects have been extensively recorded in nonneoplastic patients suffering from PCOS or metabolic diseases [3, 147] and likely may also be effective in cancer patients.

## 8. Outstanding Issues

**Figure 1:**
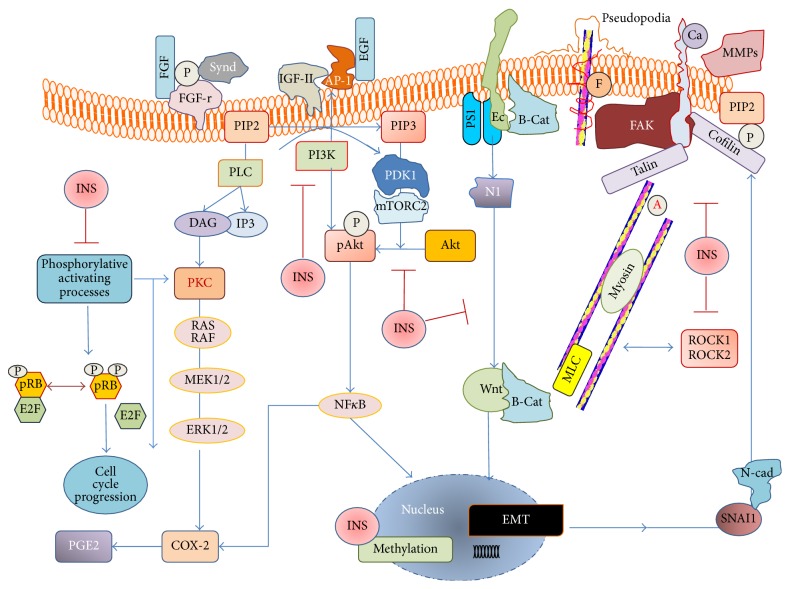
Inositol mechanisms of action in cancer cells. Inositols (INS), including InsP6 and myo-Ins, modulate a number of different critical pathways. Available data suggest that the inhibition of the phosphorylation-based (P) activation of key molecular targets represents a basic mechanism through which inositol interferes with specific biological functions, eventually ending up in delaying cell replication and in fostering apoptosis or phenotypic differentiation. (A) Inositols inhibit pRB phosphorylation, thus fostering the pRB/E2F complexes formation and blocking further progression along the cell cycle. (B) Phosphatidylinositol-4,5-bisphosphonate (PIP2) is metabolized to diacylglycerol (DAG) and Ins-trisphosphate (IP3) by phospholipase-C (PLC). Moreover, PI3K catalyzes the synthesis of PIP3 from PIP2. PIP3 is required for enabling the activation of ERK and Akt pathways. Indeed, by reducing both PI3K levels and its activity, inositols counteract the activation of the PKC/RAS/ERK pathway. Upstream of that pathway, inositols disrupt the ligand interaction between FGF and its receptor (FGF-r) by interfering with syndecan (Synd) activity as well as with the EGF-transduction processes involving IGF-II receptor and AP-1 complexes. Downstream of PI3K inhibition, Akt activation through selective phosphorylation promoted by PDK and mTORC2 is severely impaired upon inositol addition. Downregulation of both Akt and ERK leads consequently to NF-kB inhibition and reduced expression of inflammatory markers, like COX-2 and PGE2. Inositol-induced downregulation of presenilin-1 (PS1), when associated with inhibition of the PI3K/Akt pathway, counteracts the epithelial-mesenchymal transition (EMT), thus reducing Wnt-activation, *β*-catenin (*β*-cat) translocation, Notch-1, N-cadherin (N-cad), and SNAI1 release. Inositols interfere also directly with different cytoskeleton components by upregulating Focal Adhesion Kinase (FAK) and E-cadherin (Ec) and decreasing Fascin (F) and Cofilin, two main components of the pseudopodia. Reduced formation of membrane ruffling and pseudopodia, as well as inhibited release of metalloproteinases (MMPs), severely impairs both motility and invasiveness of cancer cells. This effect is reinforced by the inositol-induced inhibition on ROCK1/2 release, as well as by the decreased levels of phosphorylated Myosin Light Chain (MLC). Overall, these effects enable inositols to remodel F-actin (A) assembly and thus to reshape the cytoskeleton architecture. Blue arrow indicates promoting effect; red line with bar indicates inhibitory effect.

Both myo-Ins and InsP6 have been demonstrated to exert a wide range of anticancer effects. Namely, inositols interact with specific cancer cellular pathways, while also exerting other valuable activities at the systemic level (enhancement of immune function, antioxidant activity). The astonishing complexity of their effects (Figure 1) on so different targets allows us to consider both of them as truly “pleiotropic” agents. Moreover, as suggested by some preliminary reports, it cannot be discarded that InsP6 and myo-Ins may also play a specific epigenetic role in selected gene clusters.

### 8.1. Epigenetic Effects

In yeast, myo-Ins displays basically a repressing activity on a discrete number of genes [148], and preliminary data suggest that this is also the case in humans (personal communication). Conversely, under conditions of inositol deprivation, hundreds of genes are activated, mainly those involved in stress response pathways [149], PKC pathways [150], inositol and phospholipid biosynthesis (ISYNA1 gene) [151, 152], and glucose metabolism [153]. It is still to be investigated if the inositol-based control on gene expression should be ascribed to methylation specific activity or to other mechanisms. In addition, myo-Ins has been shown to be involved in chromatin remodeling and DNA-repair processes. Chromatin remodeling represents a critical process ruling the access for DNA-binding proteins and therefore it is required for efficient gene transcription. Mutation in genes encoding inositol polyphosphate kinases responsible for the production of InsP4, InsP5, and InsP6 impairs gene transcription* in vivo*, thus evidencing that specific inositol phosphates are required for proper transcriptional activity, thus establishing a clear link between InsPs availability and chromatin remodeling [154, 155]. Indeed, inositol phosphates are involved in gene transduction, given that depletion of InsP6, InsP7, and InsP8 by means of inositol polyphosphate multikinase inhibition impairs mRNA export from the nucleus [156]. Efficiency of gene transcription relies on DNA stability and maintenance that is primarily ensured by DNA-repair mechanisms. Homologous recombination and nonhomologous end-joining are the two main DNA-repair mechanisms frequently deregulated in a number of pathological conditions. Inositol phosphates (mainly InsP6) have been shown to foster DNA-repair processes by binding to the DNA end binding protein Ku [157]. Inositol hexakisphosphate modulates Ku dynamics [158] by interacting with a specific Ku region and, by subsequently activating the DNA-PK binding, InsP6 promotes the nonhomologous end-joining repair [159]. Furthermore, it has been shown that InsP6 binds to DNA-PK and specifically stimulates DNA-PK-dependent end-joining* in vitro* [158].

### 8.2. Synergistic Effects

There is a widespread consensus suggesting that InsP6 and myo-Ins act synergistically when added in association. That finding evidences a possible cumulative effect on selected targets or, even more likely, a complex metabolic interaction. InsP6 has indeed been demonstrated to be dephosphorylated within the cell, leading to myo-Ins or to less phosphorylated forms (namely, InsP5 and InsP4) [23, 160] which, in association with myo-Ins, may collectively modify the network of inositol-based molecules and hence a number of biochemical pathways. Moreover, a number of inositol derivatives, including lower phosphorylated forms [161, 162] and pyrophosphates [163], have been proven to exert anticancer effects. However, despite the fact that some insight has been provided by using [^3^H]InsP6 [164] or [^3^H]myo-Ins [165], we are still unable to grasp what the cellular fate of both InsP6 and myo-Ins could be after the cellular uptake. Additionally, inositol isomers may also play a significant biological role, hitherto evidenced in other diseases. For example, the association of myo-Ins and D-chiro-inositol in a proper ratio (40 : 1) has been demonstrated to be effective in polycystic ovary syndrome treatment [166], while* scyllo*-Ins is currently under scrutiny as a reliable treatment for Alzheimer and other neurological diseases [167]. It would be worth of interest to ascertain whether inositol isomers or other inositol derivatives could also exert any valuable biological effect in cancer. It is therefore mandatory to investigate thoroughly the inositol metabolomics in order to identify the main metabolic pathways of both InsP6 and myo-Ins. Furthermore, metabolomics data should be integrated with genomic pathways, thus providing the basic information required to recognize the cellular fate of therapeutically added inositols and the genomic/enzymatic targets downstream.

### 8.3. Pleiotropic Effects

Inositol and its phosphorylated derivatives (InsP6 and InsP5) interfere with several critical processes involved in the regulation of cell proliferation, apoptosis, and differentiation, including the MAPK-ERK cascade, the PI3K/Akt, and the *β*-catenin/Wnt/NF-kB pathway. The PI3K/Akt pathway has been proven to be inhibited by a wide range of inositol phosphates (InsP6, InsP5, and InsP4) [168] as well as by myo-Ins. This effect can be ascribed to several mechanisms including direct PI3K blocking (as the structure of InsP6 appears to be very similar to 3-deoxy-3-fluoro-PtdIns, a potent PI3K inhibitor) [169] or inhibiting the PI(3,4,5)P3-dependent Akt recruitment to the plasma membrane [170]. Moreover, it seems that myo-Ins, InsP6, and other inositols phosphate derivatives may modulate cell function by inhibiting several phosphorylation pathways. Activation mechanisms through phosphorylation of Ras, mitogen-activated protein kinases (MAPK), protein kinase C (PKC), PI3K, and activating-protein-1 (AP-1) are indeed downregulated by inositols via a direct control of protein phosphorylation. InsP6 inhibits the phosphorylation-induced activation of ERK and JNK activity in a number of cancer types [75, 82, 171]. InsP6 selectively activates two distinct isoforms of PKC: PKC-*ε* and PKC-*δ*. PKC-*ε* is required for insulin secretion and primes Ca^2+^-induced exocytosis in pancreatic *β*-cells upon InsP6 stimulation [172]. PKC-*δ* activity is increased severalfold after InsP6 addition, and that increase leads subsequently to enhanced release of p27, thus blocking cell cycle progression in breast cancer cells [36]. Phosphorylation of specific residues seems to be a widely used mechanism in nature for activating specific molecular effectors, while dephosphorylating performed by phosphatases (like PTEN [173], SHIP [174], or inositol polyphosphate phosphatases [175]) represents a general inhibitory tool for counteracting the same pathways. Therefore, the complexity of the inositol metabolism stands out in the midst of the even more complex field of enzymatic regulation and it is quite impossible to deal with this complexity only relying on the rules provided by the old-fashioned reductionist model. On the contrary, a systems biology approach [176] is mandatory to efficiently grasp the interwoven inositol network.

## 9. Conclusion

Myo-inositol and its derivatives, among which InsP6 occupies a relevant place, have been shown to play many biological functions, including modulation of cell cycle progression, apoptosis, and differentiation. During the last decade, evidence is mounting that inositol acts on both cytosolic and nuclear targets in enabling cells to successfully cope with many different stressors. Indeed, the inositol network seems to display a key role during developmental processes and cellular differentiation, as demonstrated by studies carried out on oocyte maturation and embryo development [177, 178].

Available results suggest that the combination InsP6+myo-Ins may be most effective to move forward in the future. It can be hypothesized that this association may enact the release of low-phosphorylated inositol derivatives (InsP5, InsP4, InsP3, and InsP2), which in turn may trigger specific effects. Alternatively, InsP6 and myo-Ins may target the same molecular mechanisms or enzymatic pathway displaying true synergistic (rather than additive) effects. However, until a metabolomic profile of added myo-Ins will be available, hypotheses on the synergistic effect of InsP6 and myo-Ins are at best presumptive.

Cancer can be considered a kind of “development gone awry” [179], in which the deregulation in the crosstalk among cells and their microenvironment plays a relevant role. Given that inositol participates in the cell-stroma interplay by modulating metalloproteinases, E-cadherin, focal kinase complexes, and many other cytoskeletal components, it can be hypothesized that inositol and its derivatives may counteract cancer-related processes by specifically acting at this level, that is, by restoring a “normal” cell-stroma relationship. Studies in this field are therefore urgently warranted in order to deepen our understanding of inositol mechanisms on cancer.
